# The latent structure of depressive symptoms across clinical high risk and chronic phases of psychotic illness

**DOI:** 10.1038/s41398-019-0563-x

**Published:** 2019-09-16

**Authors:** Teresa Vargas, Anthony O. Ahmed, Gregory P. Strauss, Cassandra M. Brandes, Elaine F. Walker, Robert W. Buchanan, James M. Gold, Vijay A. Mittal

**Affiliations:** 1 Northwestern University Department of Psychology, Evanston, IL 60208 USA; 2 Weill Cornell Medical College, New York, NY 10065 USA; 3 University of Georgia, Athens, GA 30602 USA; 4 Emory University, Atlanta, GA 30322 USA; 5 University of Maryland, Baltimore County, MD 20742 USA; 6 Northwestern University Department of Psychology, Department of Psychiatry, Department of Medical Social Sciences, Institute for Policy Research, and Institute for Innovations in Developmental Sciences, Evanston, IL 60208 USA

**Keywords:** Schizophrenia, Human behaviour

## Abstract

Depressive symptoms are highly prevalent in psychotic populations and result in significant functional impairment. Limited knowledge of whether depressive symptoms are invariant across stages of illness curtails our ability to understand how these relate to illness progression. Clarifying the latent structure of depressive symptoms across stages of illness progression would aid etiological conceptualizations and preventive models. In the present study, one-factor (including all items) and two-factor (depression/hopelessness and guilt/self-depreciation) solutions were specified through confirmatory factor analysis (CFA). Measurement invariance analyses were undertaken across schizophrenia (SCZ; *n* = 312) and clinical high-risk (CHR; *n* = 175) groups to estimate whether the same construct is being measured across groups. Clinical correlates of the factors were examined. Results indicated that CHR individuals had a greater proportion of mood disorder diagnoses. Metric invariance held for the one-factor solution, and scalar invariance held for the two-factor solution. Notably, negative symptoms did not correlate with depressive symptoms in the SCZ group, though strong correlations were observed in CHR individuals. Positive symptoms were comparably associated with depressive symptoms in both groups. Results suggest depressive symptoms are more prevalent in CHR individuals. Targeting these symptoms may aid future efforts to identify risk of conversion. Further, some depressive symptoms may be systematically more endorsed in CHR individuals. Separating into depression/hopelessness and guilt/self-depreciation scores may aid comparability across stages of illness progression, though this issue deserves careful attention and future study.

## Introduction

Depressive symptoms are highly prevalent in psychotic populations, in both first episode and chronic phases of illness^1,2^. These have been closely linked to psychotic symptom severity, distress, and content, as well as to symptom development, illness prognosis, and relapse^3^.

Importantly, they have also been associated with poor psychological well-being and functional outcomes^3,4^. There is evidence that clinical high-risk (CHR) individuals (i.e., those showing emergent attenuated positive symptoms and corresponding cognitive/functional decline, that are at imminent risk for transitioning to a psychotic disorder) also exhibit high rates of depressive symptoms^5–9^; these symptoms have been associated with poor clinical outcomes in this group^10–12^. CHR syndromes are notable in that only a minority (an estimated 10–35%) of individuals go on to eventually convert to a psychotic disorder^6,13^. In addition, rates of depression among this population may be higher than in schizophrenia^7^. Thus, it is critical to understand depressive symptoms in the context of the broader psychosis spectrum, including CHR individuals. However, it is currently unclear whether depressive phenomenology manifests similarly across groups (e.g. with regard to factor structure, symptom presentation, frequency, severity, and trajectory). Determining whether the latent structure of depressive symptoms is similar across stages of illness progression is paramount to informing efforts aimed at intervention and prevention, as differences in presentation across phases of illness would require different target approaches and conceptualizations.

Depressive symptoms within psychosis spectrum populations play a critical role in the development, maintenance, and exacerbation of negative symptoms (i.e., reductions in motivation and emotional expression relative to one’s own demographic)^5,7–9,14^. These can be primary (i.e. idiopathic to the disorder) or secondary (i.e., the result of factors other than disease processes related to psychotic disorders, such as depression)^14^. Depression is a common secondary source of negative symptoms in those with psychotic disorders, though it is unclear whether this manifests similarly in CHR individuals^14–17^. Elucidating this is key because negative symptoms can emerge years before attenuated positive symptoms, being among the strongest predictors of conversion to a psychotic disorder^5,18–20^. One might expect depression to be a significant secondary negative symptom source in CHR individuals given that rates of depression are high in this population (e.g., 41% of CHR individuals are estimated to be diagnosed with a comorbid depressive disorder)^7^. If this is the case, there are important resulting treatment implications, as a focus on depression may be critical for prevention at the earliest stages of the prodrome and as a means of halting functional deterioration.

Differing sources of negative symptoms are also important to consider from an etiological standpoint. For example, a wide body of research suggests that primary negative symptoms are distinct and not associated with depressive symptoms in individuals with psychotic disorders^14,21–24^. Predominant theoretical models accept that there are facets that are distinct to depressive (low mood, suicidal ideation, and pessimism) and negative symptoms (alogia, blunted affect), respectively, which may account for the lack of correlation in psychotic disorders^25^. However, some models suggest there may also be some overlap in symptom presentation between the two, especially with regard to anhedonia, anergia, and avolition. Thus, identifying distinct and unique latent factors for depressive and negative symptoms among CHR individuals is a necessary avenue of investigation. This may be crucial in terms of predicting disease course: depressive symptoms have previously been found to strongly correlate with patient-rated illness severity in schizophrenia^26^. Further, schizophrenia spectrum (SCZ) patients who present with predominantly primary negative symptoms have been observed to experience greater social and occupational function deficits, despite exhibiting less severe depressive symptoms^16,27–30^. This divergence suggests that primary and secondary negative symptoms may differentially predict distinct outcomes. Taking this into consideration is particularly important in CHR populations, as the presence of distinct primary/secondary negative symptom sources has not been as well examined in this critical period.

Understanding whether primary and secondary sources of negative symptoms are distinguishable in CHR individuals (as they are in SCZ individuals) would aid in conceptualizing risk models and understanding negative symptom etiology. CHR individuals by definition exhibit attenuated psychotic features. Though a majority of them may not convert to a psychotic disorder, determining whether secondary negative symptoms (i.e. depression) manifest similarly (with regard to factor structure, symptom presentation, frequency, severity, and trajectory) to chronic psychosis individuals in this group would increase our understanding of risk and negative symptom etiology. Perhaps primary negative symptoms emerge only after psychotic disorder onset, or perhaps attenuated primary negative symptoms emerge beforehand, at the CHR stage. Or, perhaps only secondary negative symptoms (i.e. depressive symptoms) are observable before psychotic disorder onset. These questions have yet to be definitively answered and establishing the latent structure of depressive symptoms across groups is a necessary first step. Further, negative symptoms separate into volitional and expressive factors in adults with psychotic disorders and CHR youth^20,31–33^. Therefore, it will also be important to determine whether depressive symptoms have different patterns of correlations with these factors, which are thought to have unique pathophysiological mechanisms^34^.

The distinct presence of primary and secondary negative symptoms is well-established in chronic stages of psychotic illness. This is not the case for CHR populations. Determining whether primary and secondary sources of negative symptoms manifest similarly in CHR individuals is crucial for etiological conceptualizations, intervention, and prevention treatments. The present investigation used confirmatory factor analysis (CFA) to compare competing models regarding the latent structure of depressive symptoms in psychotic disorders and CHR individuals. The Calgary Depression Scale for Schizophrenia (CDSS), a commonly implemented clinical rating scale, was used to address these questions^35–37^. While it is widespread clinical and research practice to calculate a single total score for the CDSS, a range of factor solutions have been found, with a two-factor solution receiving the greatest support in the empirical literature (with factors reflecting guilt and depression/hopelessness)^22–24^. In CHR populations, only a single study has examined the factor structure of the CDSS to date. The authors also found a two-factor solution, with separate dimensions for guilt/self-depreciation and depression/hopelessness^38^. However, to date, no studies have formally tested factorial invariance between the psychotic disorder and CHR samples for either a single factor or multiple-factor solutions. Thus, it is unclear whether the latent structure of depressive symptoms is comparable across phases of illness.

First, a uni-dimensional model was evaluated given the common practice of calculating a single CDSS total score. Second, a more complex model indicating two dimensions consisting of items related to depression/hopelessness and guilt/self-depreciation was tested, in line with previous studies^23,38^. To examine whether previous literature finding weak correlations with negative symptoms in schizophrenia populations would also apply to CHR populations, depression sum scores within diagnoses were correlated with positive and negative symptoms^21,22,24^. Finally, given that negative symptoms separate into volition and expression factors in SCZ patients, correlations were explored between these specific dimensions^20,31–33^. It was hypothesized that the two-factor solution would indicate a better fit than the one-factor solution, given that this two-factor structure has been found across CHR and SCZ groups. Further, it was hypothesized that positive symptoms would correlate with depressive symptoms, and that negative symptoms would not correlate with depressive symptoms in the SCZ group. We were agnostic as to the relationship between negative symptoms and depressive symptoms in CHR individuals.

## Methods

### Participants

The present study is a well-powered, multi-site collaboration, spanning across four collection sites using archival data^39^. SCZ inpatient and outpatient individuals (*n* = 312) who met DSM criteria for schizophrenia (*n* = 258) or schizoaffective disorder (*n* = 54) were recruited at the outpatient clinics at the Maryland Psychiatric Research Center (MPRC). CHR individuals meeting criteria for a psychosis risk syndrome (described below) were recruited through the North American Prodrome Longitudinal Study (NAPLS) site at Emory University (*n* = 76), and the Adolescent Development and Preventive Treatment (ADAPT) locations at Northwestern University (*n* = 25) and University of Colorado Boulder (*n* = 74). Affective disorders were defined as follows for analytic purposes: SCZ patients with a current mood diagnosis included schizoaffective disorder, and CHR individuals with a current mood diagnosis included persistent depressive disorder, major depressive disorder, and bipolar disorder (see Table 1). All protocols were approved by respective local IRBs. Informed consent was obtained from all subjects. Participants under 18 gave written assent, in addition to their guardians giving informed consent.

**Table 1:** Demographic characteristics

	Entire sample		CHR		SCZ		*χ* ^2^	df	*p*
	*n*	%	*n*	%	*n*	%			
Sex									
Female	178	36.6	76	43.4	102	32.7	5.6	1	0.018
Male	309	63.5	99	56.6	210	67.3			
Mood Dx	107	22	53	30.3	54	17	11	1	0.001
Antipsychotics	294	60.4	26	15	268	85.9			
Race^a^							73.5	2	<0.001
White	273	56.1	104	59.4	169	54.2			
Black	149	30.6	22	12.6	127	40.7			
Other	65	13.3	49	28	16	5.1			
	M	SD	M	SD	M	SD	*t*	df	*p*
Age	32.8	14.3	19.2	3.5	40.6	12.1	−29	380.5	<0.001
Years of education			12.2	2.4	13	10	−1.5	483	0.12
Depressive symptoms^b^			6	4.8	2.5	2.8			
Positive symptoms^c^			13	4.1	10	4.9			
Negative symptoms^d^			11	6.3	7.5	3.2			
CPZ equivalent dosage^e^	—	—	139.3	68.1	676.9	514.3			

Chi-square tests and independent sample *t*-tests, when appropriate, were used to test demographic differences across groups

^a^Self-reported race

^b^Measured by the CDSS for both groups

^c^Measured by the BPRS for the SCZ group, and the SIPS for the CHR group

^d^Measured by the BPRS for the SCZ group, and the SIPS for the CHR group

^e^Dosage reported for those on neuroleptics, converted to CPZ equivalents^72^

### Measures

The CDSS was used to assess depressive symptoms as secondary sources of negative symptoms. This scale has shown excellent psychometric properties in regard to inter-rater reliability, sensitivity, specificity, and discriminant/convergent validity, as well as internal consistency in schizophrenia populations^35,36,40–44^. Further, it has been found to have advantages relative to depression rating scales not specialized for schizophrenia^21,45–47^. The presence of negative symptoms endemic to psychosis can often complicate efforts to assess depressive symptoms^15^. Notably, the CDSS was also specifically designed and validated for this purpose of separating primary negative symptoms from depressive symptoms in schizophrenia^37^, and is widely recommended for assessing the severity of depressive symptoms in psychotic populations^38,48,49^_._

In the SCZ patient sample, consensus diagnosis was established via a best-estimate approach based on psychiatric history and multiple interviews and subsequently confirmed using the SCID^50^. All patients met DSM-IV lifetime diagnostic criteria for a psychotic disorder. A clinical interview was also performed, and patients were rated on the Brief Psychiatric Rating Scale (BPRS)^51^, Scale for the Assessment of Negative Symptoms (SANS)^52^, and CDSS. Ratings were made by clinicians with multiple years of clinical experience who were trained to reliability standards of alpha > 0.80 on each scale using internal gold-standard trainings conducted monthly to prevent rater drift and ensure standardization of rating procedures.

For CHR individuals, the Structured Interview for Psychosis risk Syndromes (SIPS)^53^ was administered to diagnose a psychosis risk syndrome. The SIPS rates the severity of several attenuated psychotic symptoms on 7-point scales. Positive symptoms are rated absent (0) to psychotic (6), and negative symptoms are rated absent (0) to severe (6). For the purposes of this study, SIPS ratings ranged from 0 to 5, as ratings of 6 indicate psychotic level symptoms, which would signal the individual to be “too far along” for a CHR diagnosis. Sum scores were used to quantify positive and primary negative symptoms. In addition, the Structured Clinical Interview for DSM-IV and 5 (SCID)^54^ was used to assess for the presence of a formal psychotic disorder and/or the presence of any non-psychotic psychiatric diagnoses. Both of these measures have been used extensively in studies with CHR individuals, and previous research has shown that the SCID has excellent inter-rater reliability^55^. All interviews were conducted by experienced researchers who underwent extensive gold-standard training; Kappas of at least 0.8 for SIPS and 0.9 for psychosis risk and psychiatric diagnoses were obtained, consistent with previous lab investigations^20^.

### Data analyses

#### Preliminary analyses

Data were checked for skew using SPSS. The CDSS sum score was skewed in both groups (median = 2, interquartile range = 3 in SCZ patients and median = 5, interquartile range = 9 in CHR individuals). Given that the use of the CDSS as a total sum score in the literature assumes a one-factor solution, a one-factor model was tested including all CDSS items using CFA. As indicator variables share components, correlations were allowed between residual terms^56–58^. The Maximum Likelihood estimator was applied with robust standard errors (MLR) in order to obtain appropriate fit indices for skewed data^57^. The Comparative Fit Index (CFI), Tucker Lewis Index (TLI), Root Mean Square Error of Approximation (RMSEA), and Standardized Root Mean Square Residual (SRMR) were used to evaluate model fit. CFI and TLI values ≥0.90, RMSEA ≤ 0.10, and SRMR ≤ 0.06 were considered indicative of acceptable model fit^59,60^.

#### Multi-group CFA analyses

Measurement invariance was tested across groups using multi-group CFA, and involved four consecutive steps: configural, metric/weak, scalar/strong, and strict invariance. These steps iteratively constrain the number of factors, loadings, intercepts, and residuals to equality. At each step, the fit of the more constrained model is tested against the previous model through a chi-square difference test. For this test, an alpha of 0.05 was used as a threshold for statistical significance, in keeping with the conventional threshold. Given sample sizes of *n* > 300, a change of CFI ≤ −0.10 along with a change in RMSEA of ≤0.015 was indicative of invariance^61^.

#### Measurement invariance analyses

Configural invariance was first tested and determined whether the factor structure was similar in both diagnoses. Configural invariance testing assumes that a set of observed measures evoke the same conceptual frame of reference in each group at the most basic level^62^. That is, whether a measure evaluates a given latent construct similarly across groups. If configural invariance held, metric (weak) invariance would then be tested. Metric invariance models constrain the item factor loadings to be equal across groups. If metric invariance holds, structural relations between groups, such as factor correlations, may be examined and compared across groups^63^. In that case, scalar invariance would be tested, which constrains the intercepts to be equal across groups. This is the minimum level of invariance required to allow for the comparison of latent means and regression parameters between groups^64^. A lack of scalar invariance indicates differential item functioning between groups. If strong invariance holds, strict invariance would be tested by also constraining residuals to equality between groups. If the measure is not invariant at any level prior to strict invariance, more constrained models are not tested^63,64^.

#### Unifactorial and two-factor analyses

**Fig. 1:**
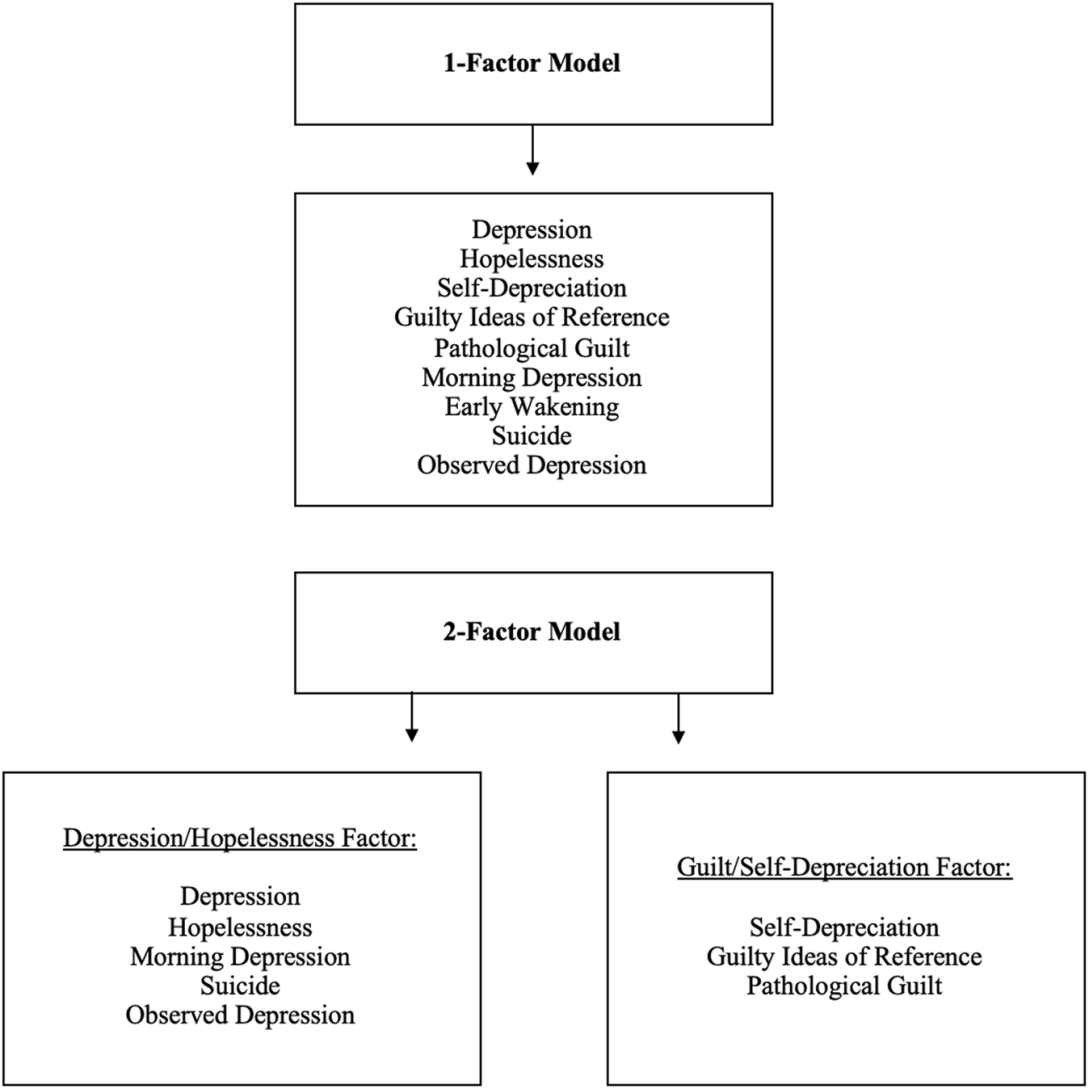
CFA models tested

A one-factor solution was first tested. Then, a two-factor solution was tested separating depression/hopelessness and guilt/self-depreciation items, in line with previous studies^21,23,38^. The first depression/hopelessness factor included five items (depression, hopelessness, morning depression, suicide, and observed depression). The second guilt/self-depreciation factor included three items (self-depreciation, guilty ideas of reference, and pathological guilt). Item 7, early wakening, showed very low or absent correlations with other items (Supplementary Tables 1 and 2). Thus, in line with other studies of two-factor models showing its removal improved internal consistency, it was excluded from analyses; see Fig. 1 for diagram of models tested^23,38^. For reliability information, please see Supplementary Material and Supplementary Table 1. CFA, measurement invariance, and reliability analyses were conducted using the R packages psych, lavaan, and semTools^57,65–67^. Finally, all analyses were re-run excluding individuals with affective disorders in the SCZ and CHR groups. This was done to determine whether results were driven by individuals with a diagnosed affective disorder, as we were also interested in depressive symptom measurement at “sub-threshold” levels not severe enough to qualify for an affective disorder diagnosis.

#### Analyses examining associations with positive and negative symptomatology

To explore clinical correlates of depressive factors across phases of psychotic illness, point-biserial correlations were used to predict CDSS sum score with presence or absence of an affective disorder diagnosis. In this vein, CDSS sum score and two factors were correlated with positive symptoms in SCZ and CHR groups using Spearman correlations (due to data skew; Pearson correlation results are similar and presented in Supplementary Table 3); this is given previous studies finding associations between positive symptom severity, illness severity, and CDSS sum score^26,49^. As a test of discriminant validity, Spearman correlations were used to test associations between negative symptoms and CDSS sum score within SCZ and CHR groups separately. Finally, associations with primary negative symptom subdimensions of volition and emotional expression (measured by the SANS and the SIPS in SCZ and CHR samples, respectively) were tested. Finally, in an exploratory fashion, Spearman correlations were run between antipsychotic dosage (among those individuals on neuroleptics), total depressive symptoms (secondary sources of negative symptoms), and total primary negative symptoms.

## Results

Expectedly (and rather unavoidably for any investigations comparing across CHR and SCZ stages), the SCZ group had a greater proportion of males, higher neuroleptic usage, and greater age compared to CHR individuals. A large majority of the CHR sample was neuroleptic free (85%), while a majority of the SCZ sample was medicated (14% neuroleptic free). There were no significant associations between antipsychotic dosage, depressive symptoms, and negative symptoms across groups (*p* = 0.08–0.76), except for an association between total CDSS score and antipsychotic dosage in the SCZ group (*r* = 0.12, *p* = 0.04). This could be due to increased illness severity in those with greater neuroleptic dosages, which has been correlated with depressive symptoms in SCZ^26^. The prevalence of depressive diagnoses was roughly 22% across groups, with a higher proportion in the CHR group (see Table 1). In terms of reliability of the scale as a whole, using a confirmatory one-factor model with SEM across the whole sample, total = 0.91. For the CHR group, *ω* total = 0.86. For the SCZ group, *ω* total = 0.8 (see Supplementary Table 3).

### Measurement invariance

The one-factor CFA model showed an adequate fit, Robust *χ*^2^ = 69.10, df = 27, *p* < 0.001, Robust TLI = 0.93, CFI = 0.95, RMSEA = 0.07, SRMR = 0.04. When individuals with an affective disorder were removed from analysis (remaining *n* = 258 for SCZ, *n* = 122 for CHR), the one-factor model remained an adequate fit (Robust TLI = 0.91, CFI = 0.93, RMSEA = 0.08, SRMR = 0.05; see Table 2). The model met for metric invariance, indicating that the loadings were similar between groups.

**Table 2:** Measurement invariance analyses

Model	*χ* ^2^	df	*χ* ^2^	*p*	CFI	CFI	RMSEA	RMSEA
Thresholds						≤−0.010		≤0.015
**One-factor model**								
Configural invariance	103.99	54	—	—	0.94	—	0.05	—
Metric invariance	118.21	62	12.59	0.13	0.93	0.01	0.05	0
Scalar invariance	137.36	70	19.16	0.01	0.91	0.02	0.05	0
**Two-factor model**								
Configural invariance	104.43	40	—	—	0.94	—	0.06	—
Metric invariance	122.20	47	9.58	0.21	0.94	0.003	0.06	0.003
Scalar invariance	135.24	54	12.54	0.08	0.93	0.01	0.06	<0.001
Strict invariance	312.76	62	66.61	<0.001	0.76	0.168	0.1	0.04

The CFA specifying a two-factor structure was a good fit, Robust *χ*^2^ = 44.29, df = 19, *p* = 0.001, TLI = 0.94, CFI = 0.96, RMSEA = 0.05, SRMR = 0.04. The two-factor model remained an adequate fit when schizoaffective disorder individuals were excluded (TLI = 0.92, CFI = 0.95, RMSEA = 0.08, SRMR = 0.05). The correlation between the two factors was *r* = 0.90. The model met for scalar invariance, indicating that the loadings and intercepts were similar between groups (see Tables 2 and 3).

**Table 3:** Intercepts, latent variables, and variances for one- and two-factor models for CHR and SCZ groups

		CHR			SCZ	
	Intercepts	Variances	Latent variables	Intercepts	Variances	Latent variables
**One-factor model**						
Depression	1.09	0.42	1.0	0.47	0.24	1.0
Hopelessness	0.57	0.28	0.73	0.28	0.20	0.87
Self-depreciation	0.86	0.61	0.93	0.33	0.28	0.85
Guilty ideas of reference	0.47	0.56	0.35	0.18	0.21	0.40
Pathological guilt	0.77	0.52	0.79	0.39	0.30	0.61
Morning depression	0.57	0.24	0.61	0.26	0.21	0.77
Early wakening	0.57	0.76	0.21	0.21	0.28	0.02
Suicide	0.32	0.26	0.48	0.1	0.14	0.25
Observed depression	0.52	0.27	0.71	0.24	0.14	0.63
**Two-factor model: depression/hopelessness factor**						
Depression	1.09	0.39	1.00	0.47	0.24	1.0
Hopelessness	0.57	0.27	0.72	0.28	0.20	0.87
Morning Depression	0.57	0.23	0.60	0.26	0.21	0.77
Suicide	0.32	0.27	0.46	0.10	0.14	0.25
Observed Depression	0.52	0.26	0.70	0.24	0.14	0.63
**Two-factor model: guilt/self-depreciation factor**						
Self-depreciation	0.86	0.53	1.00	0.33	0.27	1.00
Guilty ideas of reference	0.47	0.53	0.42	0.18	0.21	0.48
Pathological guilt	0.77	0.43	0.87	0.39	0.29	0.72

### Correlations between depression, symptoms and affective disorder diagnosis

In CHR individuals, there were strong correlations between depressive symptoms and primary negative symptoms, which was not the case for SCZ. For both CHR and SCZ groups, there were correlations between depressive symptoms and positive symptoms (see Table 4). As expected, having an affective disorder diagnosis predicted higher CDSS sum score for SCZ [*r* = 0.22, *p* < 0.001] and CHR [*r* = 0.42, *p* < 0.001] groups. Finally, for the SCZ sample neither the volition [*r* = 0.07, *p* = 0.27] nor the emotional expression [*r* = −0.01, *p* = 0.83] domains correlated with total depressive symptoms. This was also the case for the depression/hopelessness and guilt/self-depreciation factors (*p* = 0.12–0.89). For the CHR sample both the volition [*r* = 0.47, *p* < 0.001] and the emotional expression [*r* = 0.17, *p* = 0.03] domains correlated with total depressive symptoms. This was also the case for depression/hopelessness ([*r* = 0.46, *p* < 0.001] for volition and [*r* = 0.19, *p* = 0.01] for emotion expression) and partially for guilt/self-depreciation ([*r* = 0.32, *p* < 0.001] for volition and [*r* = 0.09, *p* = 0.23] for emotion expression).

**Table 4:** Spearman correlations between depressive symptoms assessed by the CDSS and symptoms in CHR and SCZ groups

			CHR			SCZ	
		Sum score	Guilt/self- depreciation	Depression/hopelessness	Sum score	Guilt/self-depreciation	Depression/hopelessness
	**Positive symptoms**						
	SIPS	*r* *=* 0.31**	*r* *=* 0.31**	*r* *=* 0.23**			
		*p* *<* 0.001	*p* *<* 0.001	*p* *=* 0.003			
	BPRS				*r* *=* 0.24**	*r* *=* 0.25**	*r* *=* 0.15**
					*p* *<* 0.001	*p* *<* 0.001	*p* *=* 0.008
	SIPS	*r* *=* 0.45**	*r* *=* 0.34**	*r* *=* 0.43**			
		*p* *<* 0.001	*p* *<* 0.001	*p* *<* 0.001			
	**Negative symptoms**						
	BPRS				*r* *=* −0.03	*r* *=* −0.01	*r* *=* 0.02
					*p* *=* 0.66	*p* *=* 0.81	*p* *=* 0.68
	SANS				*r* *=* 0.05	*r* *=* 0.08	*r* *=* 0.05
					*p* *=* 0.47	*p* *=* 0.20	*p* *=* 0.43

*p *<* 0.05; **p < 0.01

## Discussion

The present investigation is the first to apply a measurement invariance approach to the critical question of secondary influences on negative symptoms (i.e. depressive symptoms) across stages of severity within the psychosis spectrum. The one-factor model demonstrated metric/weak invariance across SCZ and CHR groups, suggesting that depressive symptoms are systematically more likely to be present in CHR individuals. Notably, the two-factor solution that was explored separating depression and guilt/self-depreciation factors met for scalar invariance (*p* = 0.08; see Table 2), indicating a better fit across groups than the one-factor solution. Further, excluding individuals with an affective disorder diagnosis did not alter fit substantially. Associating depression measures with positive and negative symptom scales allowed for exploring critical questions regarding clinical correlates of depressive factors across phases of psychotic illness. Of particular interest, depressive symptoms were highly correlated with negative symptoms in CHR individuals, but not significantly correlated with negative symptoms across two separate scales in SCZ patients. In summary, this investigation suggests that sum scores/one-factor models for depression may not be comparable in magnitude across the psychosis spectrum. In addition, current measures of negative symptoms in CHR individuals may not be fully distinguishing secondary negative symptoms (depression) from primary negative symptoms (i.e. symptoms idiopathic to the disorder) prior to psychosis onset. It is also a possibility that primary and secondary negative symptoms are not distinguishable at this stage. However, it may prove useful to separate depression/hopelessness and guilt/self-depreciation factors if one is to compare across stages of illness progression. Present results are critical for etiological conceptualizations, as well as for informing treatment and prevention efforts.

Findings with regard to measurement invariance of a one-factor model for depression yielded a lack of similarity in intercepts. This was apparent for all items, though particularly so for depression (item 1) and hopelessness (item 2), such that compared to the SCZ group, the CHR group had a higher likelihood of endorsing these items (see Tables 2 and 3). Further, in the present sample, CHR individuals had a significantly greater proportion of individuals diagnosed with a depressive disorder. Results support the literature, namely the notion that depressive disorders are highly prevalent in the CHR period, with estimates as high as almost half of the population having a diagnosis^6,7,11,18^. However, excluding individuals diagnosed with a mood disorder did not alter the fit of the one-factor model. Thus, findings suggest that even sub-threshold mood symptoms may be systematically more present in CHR individuals versus SCZ patients. Therefore, sum depression scores may not be directly comparable in frequency or intensity across stages of psychotic illness.

The tested two-factor structure yielded compelling results, adding complexity to interpretation (see Tables 2 and 3). Consistent with prior studies in schizophrenia^22–24^ and CHR^38^ populations, support for a two-factor solution was found, with factors of depression/hopelessness and guilt/self-depreciation. This met for scalar invariance, constituting a better fit than that of the one-factor structure. However, the model did not meet for strict invariance, meaning the residuals were not equal across groups. Demographic differences across groups may be contributing to this lack of equality in residuals. Notably, associations of depression with negative symptom measures remained non-significant in SCZ patients, and strong for both factors in CHR individuals. Taken together, these results suggest that the field may benefit from separating depression/hopelessness and guilt/self-depreciation items, as this may aid comparability across CHR and psychotic disorder groups. However, it is worth emphasizing that the model only narrowly met for scalar invariance (*p* = 0.08; see Table 2). Further, given the latent factor correlation was rather high (*r* = 0.9), perhaps the distinction is not substantial between the models, despite metrics indicating a slightly better fit. Thus, concerns remain regarding possible systematic differences in item endorsement between groups.

Clinical correlates of depressive factors across phases of psychotic illness were explored. Of note, there were strong positive associations between depressive symptoms and positive symptoms, in line with previous studies finding depression to be associated with illness severity^26^, course, and prognosis^3^. Additional findings raise relevant considerations with regard to differentiating sources of negative symptoms (primary versus secondary) in CHR populations. Barring one highly informative investigation suggesting differential trajectories of primary versus secondary negative symptoms in CHR individuals^5^, this question has been largely understudied. In the present sample, negative symptoms as assessed by the SIPS were highly correlated with depressive symptoms in CHR individuals. This is in contrast to the SCZ group, where depressive symptoms were not associated with negative symptoms across two different scales (the SANS and the BPRS). Results in the SCZ group are consistent with the wider literature finding a lack of correlation between secondary sources of negative symptoms (depression) and primary negative symptoms^21–24^. Though further research is needed, results suggest that secondary negative symptoms such as depression may not only be more prevalent in CHR populations, but they may also not be as easily differentiated from primary negative symptoms relative to SCZ patients^5,15,16,68^. It is also key to note, however, that because depressive symptom endorsement frequencies were much higher in CHR individuals, this could have increased variance, contributing to the strength of the correlations.

Results suggest there is a possibility that primary negative symptoms are not meaningfully distinguishable from secondary negative symptoms at the CHR stage of illness progression. This is a critical question to address, especially given that the literature has yet to reach a conclusion on when primary negative symptoms emerge^5^. Future investigations will be needed to further clarify whether the distinction of primary and secondary negative symptoms is meaningful at CHR stages of illness progression^68^. The literature suggests that whereas secondary negative symptoms may change across illness trajectory, primary negative symptoms may be relatively stable^5,15^. Research attempting to differentiate primary and secondary negative symptoms in CHR individuals is rather limited, though a previous study has reported differences in trajectories between primary and secondary symptoms in this group, suggesting these may differentially predict risk of conversion to psychosis^5,69^. If sources of negative symptoms are distinguishable in CHR individuals, perhaps those CHR participants that have higher primary negative symptoms at baseline are more likely to convert, or alternatively, those whose secondary negative symptoms increase over time could be more likely to convert. It could also be of great benefit to determine whether those who exhibit greater primary negative symptoms are at higher risk of conversion relative to those that present chiefly with secondary negative symptoms. Present study results highlight the need for future investigations interested in the phenomenology of depression and negative symptoms in CHR individuals, in order to develop novel measurement techniques aiming to differentiate depressive from primary negative symptoms in this group.

Previous psychometric studies in CHR individuals have found volition and emotional expression as separate factors as assessed by the SIPS (linked to distinct functional outcomes, consistent with findings in chronic psychosis), with the emotional expression factor comprising emotional expression, emotional experience, and social anhedonia items, and the volition factor comprising occupational functioning and avolition^20^. In the present study, exploratory correlations of depression with these negative symptom dimensions yielded a lack of association in SCZ patients. In CHR individuals, however, significant associations were found in the same direction for both dimensions, though the strength of the association for volition was greater than that found for emotion expression. Perhaps current measures of negative symptoms in CHR groups (i.e. SIPS) are picking up more overlapping components of volition-related symptoms and depressive etiology. Future studies are needed to determine whether volition in CHR individuals manifests in a systematically distinct way from SCZ patients, as well as whether this dimension differentially predicts illness progression.

Unfortunately, in the present study sample sizes did not permit forming primary negative symptom subgroups in each population. Further, standardized means of delineating secondary negative symptoms and their specific sources on any rating scales are not available at the present time. These circumstances limit our ability to answer more refined questions with regards to negative symptom dimensions. Also, it is critical to consider that though widely used and thoroughly validated in CHR groups, extant SIPS negative symptom items contain some limitations surrounding content validity. For example, SIPS social anhedonia rating does not explicitly evaluate pleasure, while also conflating asociality, social anxiety and social skill^70^. In addition, the SIPS negative symptom domains (social anhedonia, avolition, expression of emotion, experience of emotions and self, ideational richness, and occupational functioning) do not entirely map onto the negative symptom domains identified by the NIMH for psychosis disorder individuals (anhedonia, avolition, asociality, blunted affect, alogia)^70^. This further highlights the need for scale development for negative symptoms in CHR individuals. Scales of primary negative symptoms that are invariant across CHR and chronic psychosis have yet to be developed, and so we were limited to using separate negative symptom scales for CHR and chronic psychosis groups respectively. Future studies could also benefit from combining expert ratings (as in the present study) with patient self-reported ratings. In addition, due to sample size restrictions, this investigation was unable to evaluate the roles of ethnicity, gender and culture.

We undertook a cross-sectional approach. However, a longitudinal approach examining whether measurement invariance holds across time for prodromal individuals converting to a psychotic disorder would be informative. It would also be beneficial in tracking the progression of depression along with negative symptoms. Including a first episode psychosis group would also have been greatly informative in more fully depicting the psychosis spectrum. The present investigation included primary psychotic disorder individuals (schizophrenia and schizoaffective disorder). It will be informative for future investigations to also include individuals with affective disorders with psychotic features, such as depression or bipolar disorder with psychotic features. This will allow us to broaden the breadth of representation of the psychosis spectrum, as the lack of inclusion of these individuals may have influenced current results. Given the greater prevalence of mood disorders among females^71^, future studies with larger samples allowing properly powered analyses may benefit from grouping based on gender. Future studies with available data would also benefit from examining duration of illness across groups. Finally, all chronic psychosis individuals were recruited at the same site, and CHR individuals were administered the same interviews following equivalent procedure, with no significant differences in demographic characteristics between the three recruitment sites. Nonetheless, ideally future investigations would use data from the same site in order to minimize heterogeneity.

In all, present results provide valuable information for treatment of CHR populations, as well as for increasing our understanding of the etiology of negative symptoms with relation to depressive symptoms. Further, findings offer useful measurement information for groups seeking to assess negative and depressive symptoms across the psychosis spectrum. Possible systematic differences in endorsement of depressive symptoms in CHR may suggest that depression could be an apt target for intervention at this stage of illness progression. Treating depressive symptoms early in the prodrome may aid in halting functional deterioration. Finally, the present results highlight the need for future measurement efforts addressing the question of at which stage of illness progression primary negative symptoms emerge. Further focus on this critical question may aid us in better understanding the etiological roots of psychosis and inform symptom targets for early intervention and treatment efforts.

## Supplementary information

Supplementary tables 1,2 and 3

Supplementary Table Legends
