# Supplementary material for: The latent structure of depressive symptoms across clinical high risk and chronic phases of psychotic illness
Source: Transl Psychiatry. 2019 Sep 16;9:229. doi: 10.1038/s41398-019-0563-x (PMC6746855; doi:10.1038/s41398-019-0563-x)
Supplement: Supplementary file 1 — Supplementary tables 1,2 and 3 [file 41398_2019_563_MOESM1_ESM.docx]

Supplementary Materials.

**Supplementary Table 1.** Item endorsement and reliabilities for CHR and SCZ groups.

|  | CHR | | | | SCZ | | | |
| --- | --- | --- | --- | --- | --- | --- | --- | --- |
|  | n endorsed* | % endorsed | ω _total_ loads | ω _total_ h^2^ | n endorsed* | % endorsed | ω _total_ loads | ω _total_ h^2^ |
| Depression | 116 | 66.28 | 1.0 | 1.0 | 122 | 39.10 | 1.0 | 1.0 |
| Hopelessness | 76 | 43.43 | 0.73 | 0.53 | 69 | 22.12 | 0.87 | 0.76 |
| Self-Depreciation | 85 | 48.57 | 0.93 | 0.87 | 77 | 24.68 | 0.85 | 0.71 |
| Guilty Ideas of Reference | 56 | 32 | 0.35 | 0.12 | 44 | 14.10 | 0.40 | 0.16 |
| Pathological Guilt | 83 | 47.43 | 0.79 | 0.62 | 102 | 32.69 | 0.60 | 0.37 |
| Morning Depression | 84 | 48 | 0.61 | 0.37 | 65 | 20.83 | 0.77 | 0.60 |
| Early Wakening | 60 | 34.29 | 0.21 | 0.04 | 50 | 16.03 | 0.02 | 0 |
| Suicide | 43 | 24.57 | 0.48 | 0.23 | 24 | 7.69 | 0.25 | 0.06 |
| Observed Depression | 66 | 37.71 | 0.71 | 0.50 | 69 | 22.12 | 0.63 | 0.40 |

*endorsed with scores of 1, 2 or 3.

**Supplementary Table 2.** Correlation matrix for **CHR (bolded, left of diagonal)** and *SCZ (italicized, right of diagonal)* populations.

|  | Depression | Hopelessness | Self-Depreciation | Guilty Ideas of Reference | Pathological Guilt | Morning Depression | Early Wakening | Suicide | Observed Depression |
| --- | --- | --- | --- | --- | --- | --- | --- | --- | --- |
| Depression |  | *0.40* | *0.35* | *0.15* | *0.28* | *0.49* | *0.03* | *0.12* | *0.44* |
| Hopelessness | **0.60** |  | *0.42* | *0.27* | *0.27* | *0.38* | *-0.08* | *0.22* | *0.37* |
| Self-Depreciation | **0.43** | **0.51** |  | *0.21* | *0.22* | *0.31* | *-0.04* | *0.21* | *0.37* |
| Guilty Ideas of Reference | **0.17** | **0.13** | **0.30** |  | *0.28* | *0.17* | *0.00* | *0.14* | *0.23* |
| Pathological Guilt | **0.44** | **0.46** | **0.47** | **0.38** |  | *0.25* | *-0.03* | *0.17* | *0.26* |
| Morning Depression | **0.61** | **0.42** | **0.46** | **0.13** | **0.42** |  | *0.08* | *0.15* | *0.30* |
| Early Wakening | **0.13** | **0.00** | **0.16** | **0.22** | **0.17** | **0.07** |  | *-0.00* | *0.07* |
| Suicide | **0.32** | **0.45** | **0.42** | **0.29** | **0.36** | **0.35** | **0.19** |  | *0.17* |
| Observed Depression | **0.56** | **0.49** | **0.44** | **0.23** | **0.42** | **0.50** | **0.13** | **0.49** |  |

**Supplementary Table 3.** Pearson correlations between depressive symptoms assessed by the CDSS and symptoms in CHR and SCZ groups.

|  | | CHR | | | SCZ | | |
| --- | --- | --- | --- | --- | --- | --- | --- |
|  |  | Sum score | Guilt/Self-depreciation | Depression/Hopelessness | Sum score | Guilt/Self-depreciation | Depression/Hopelessness |
| Positive symptoms | SIPS | *r* = 0.30  *p* < 0.001 | *r* = 0.28  *p* < 0.001 | *r* = 0.23  *p* < 0.001 |  |  |  |
|  | BPRS |  |  |  | *r* = 0.26  *p* < 0.001 | *r* = 0.29  *p* < 0.001 | *r* = 0.22  *p* < 0.001 |
| Negative symptoms | SIPS | *r* = 0.43  *p* < 0.001 | *r* = 0.33  *p* < 0.001 | *r* = 0.42  *p* < 0.001 |  |  |  |
|  | BPRS |  |  |  | *r* = -0.02  *p* = 0.78 | *r* = 0.00  *p* = 0.96 | *r* = 0.01  *p* = 0.88 |
|  | SANS |  |  |  | *r* = 0.08  *p* = 0.22 | *r* = 0.10  *p* = 0.10 | *r* = 0.08  *p* = 0.21 |
