# Supplementary material for: The latent structure of depressive symptoms across clinical high risk and chronic phases of psychotic illness
Source: Transl Psychiatry. 2019 Sep 16;9:229. doi: 10.1038/s41398-019-0563-x (PMC6746855; doi:10.1038/s41398-019-0563-x)
Supplement: Supplementary file 2 — Supplementary Table Legends [file 41398_2019_563_MOESM2_ESM.docx]

Supplementary Table 1. Item endorsement and reliabilities for CHR and SCZ groups. Supplementary Table 2. Pearson correlations between depressive symptoms assessed by the CDSS and symptoms in CHR and SCZ groups.
